# Supplementary material for: Meningococci drive host membrane tubulation to recruit their signaling receptors
Source: Nat Commun. 2025 Nov 25;16:10433. doi: 10.1038/s41467-025-65436-1 (PMC12647851; doi:10.1038/s41467-025-65436-1)
Supplement: Supplementary file 1 — Supplementary Information [file 41467_2025_65436_MOESM1_ESM.pdf]

Supplementary figure 1

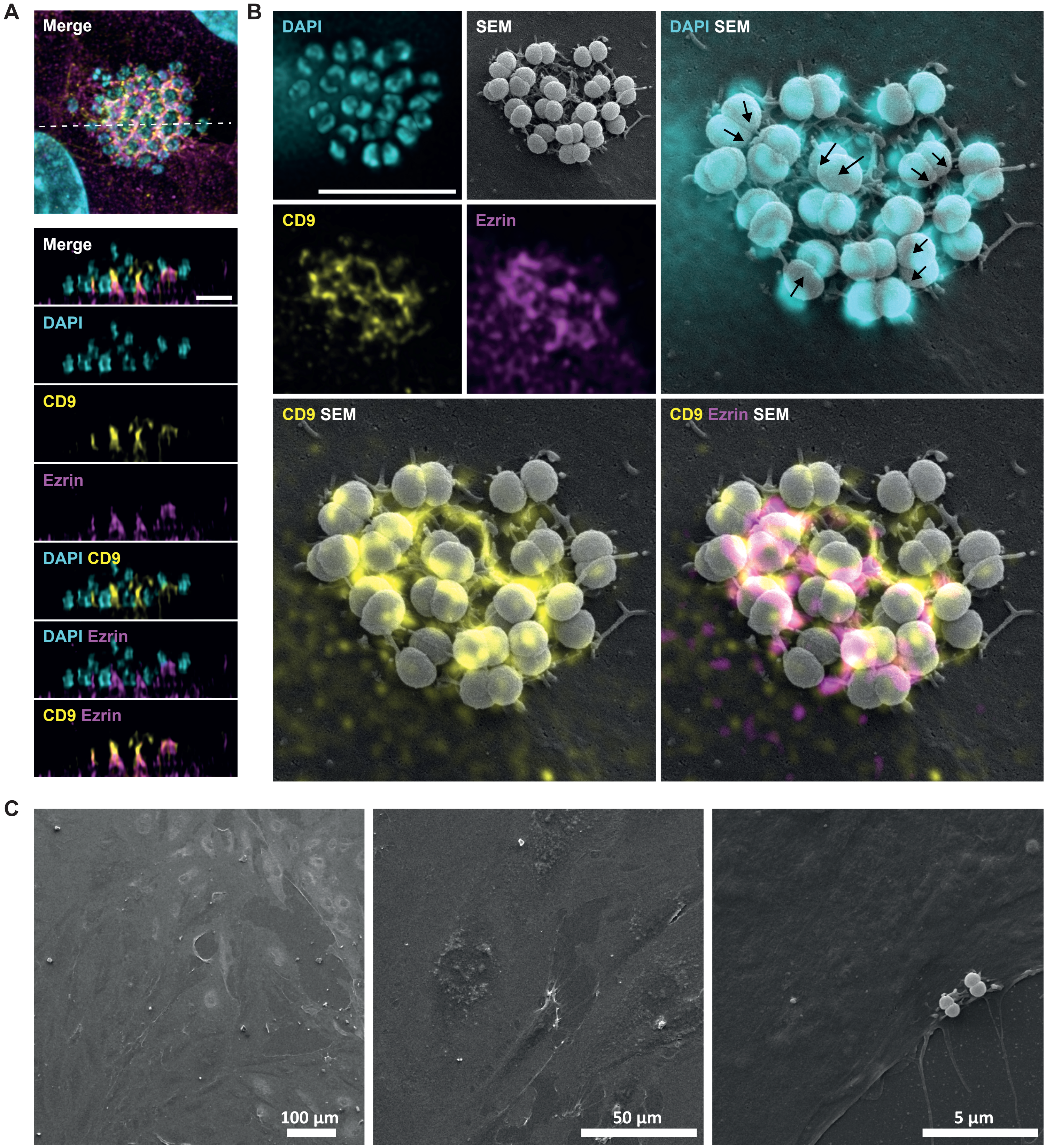

Supplementary figure 1: Recruitment of the endogenous membrane curvature-sensitive CD9 tetraspanin to meningococcal colonies
(A) Complementary images from Figure 1A. Same representative confocal microscopy images with deconvolution of a wild type meningococcal colony adhering to human endothelial cell EA.hy926 than in Figure 1. CD9 (membrane marker, yellow) and ezrin (signaling marker, magenta) were immunostained and DAPI was used to reveal cell and bacterial DNA (cyan). Location of the orthogonal slicing (white dashed line) and different merge combinations for better appraisal of DAPI, CD9 and Ezrin staining relative to one-another. B) Complementary images from Figure 1B. Representative correlative light and electron microscopy images obtained after repositioning confocal imaging with deconvolution with scanning electron microscopy (SEM). CD9 and ezrin were immunostained and DAPI was used to reveal cell and bacterial DNA. Black arrows indicate shifting of the bacteria between the immunofluorescence and SEM stages. CD9 and Ezrin immunofluorescence images superposition to the SEM image was done after the repositioning with the Icy software and EC-CLEM plugin. Full image and different merge combinations for better appraisal of DAPI, CD9 and Ezrin staining and SEM relative to one-another. Scale bars 5 µm. (C) Scanning electron microscopy of endothelial cells at increasing zoom (left to right). The uninfected cells (first and second images) are devoid of TMS. The infected cell observed in the far right picture only exhibits membrane deformation at the location of meningococcal adhesion. The three images were taken separately.

Supplementary figure 2

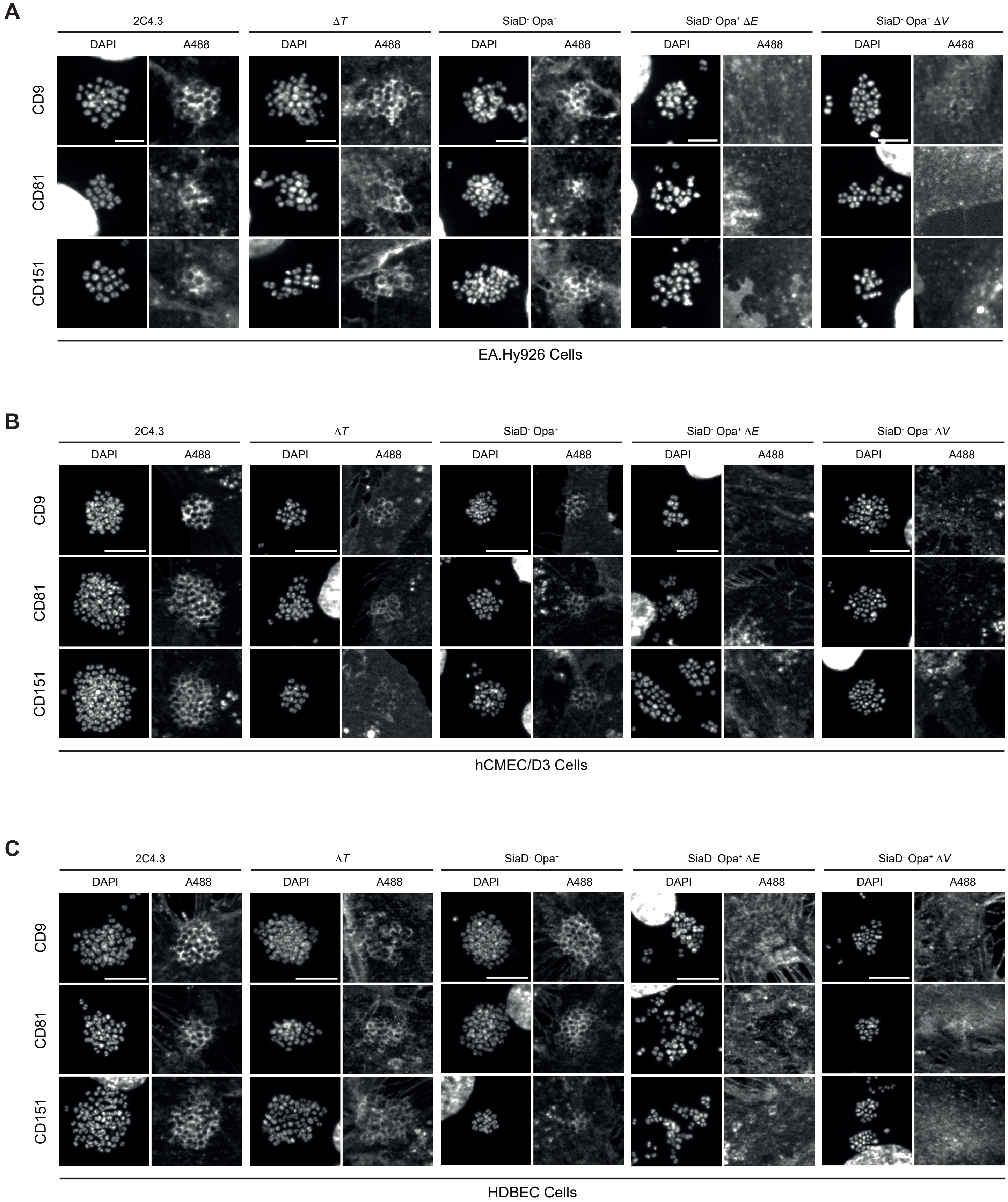

Supplementary figure 2: Tetraspanin rectruitment to wild type and mutant meningococcal colonies infecting various types of endothelial cells

Complementary images from Figure 1. Representative immunofluorescence imaging of endothelial cells (A) EA.hy926, (B) hCMEC/D3 and (C) HDBEC, immunostained for endogenous tetraspanins CD9, CD81, CD151 (three independent experiments). The cells were infected with wild-type (2C4.3) and its derivative mutant for pilT (ΔT) or the capsule mutant expressing OpaB adhesin (SiaD- Opa+) and its derivatives mutant for pilE (ΔE) or for pilV (ΔV). Z stack sum projection. Scale Bar 5μm (A) and 10 μm (B and C).

Supplementary figure 3

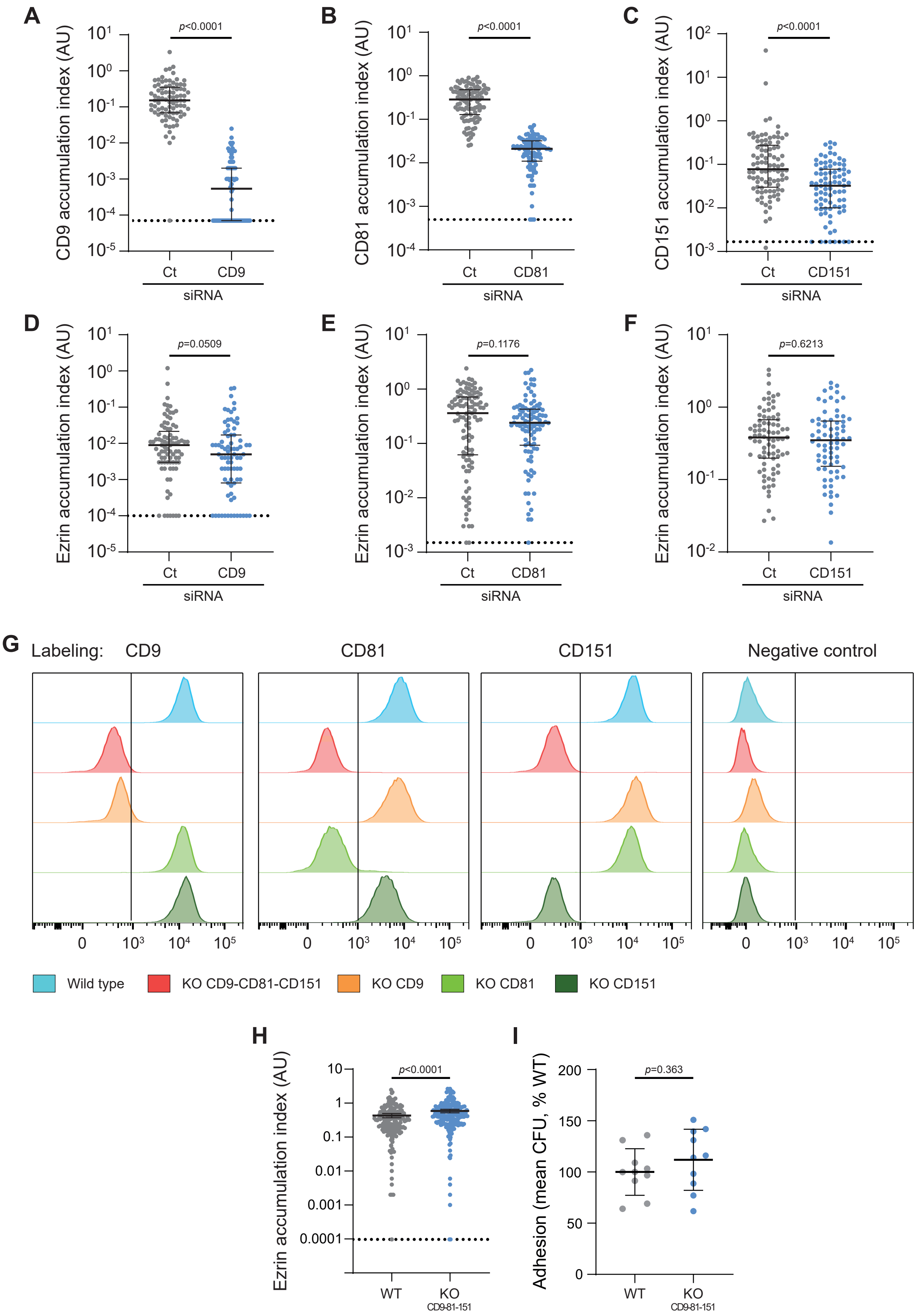

Supplementary figure 3: Phenotypic effect of tetraspanin KO on endothelial cell infection

(A-F) Infection with wild type 2C4.3 strain of EA.hy926 endothelial cells treated with siRNA targeting CD9 (A and D), CD81 (B and E) or CD151 (C and F). Immunofluorescence quantification of the accumulation of the targeted tetraspanin (A-C, respectively) or Ezrin (D-F) below the bacterial colonies, normalized with the volume of DAPI fluorescence of the corresponding colonies. Dashed line is the detection threshold. Three experiments were pooled. Data are medians ± interquartile range. (G, H, I) Phenotypes of triple CRISPR knockouts EA.hy926 cells. (G) Successive CRISPR knockouts were conducted in cells through lentivirus transfection to result in cell lines depleted of tetraspanins CD9, CD81, CD151 and all three. The purity of the KOs was assessed for each cell line through flow cytometry. Negative controls are stained with secondary antibody alone. The vertical black bar is a visual aid to separate assessed negative (left) or positive (right) staining. (H) Quantification of Ezrin accumulation in triple KO cells and below meningococcal colonies, normalized with DAPI fluorescence (Ezrin accumulation index). The dashed line represents the detection threshold. Three experiments were pooled. Data are presented as means ± 95% confidence intervals; Mann Whitney test. (I) Adhesion experiment of *N. meningitidis* strain 2C4.3 on wild type or triple KO cells. CFU mean percentage of the adhesion on wild type cells. Three experiments were pooled. Data are presented as means ± SD; Mann-Whitney test.

Supplementary figure 4

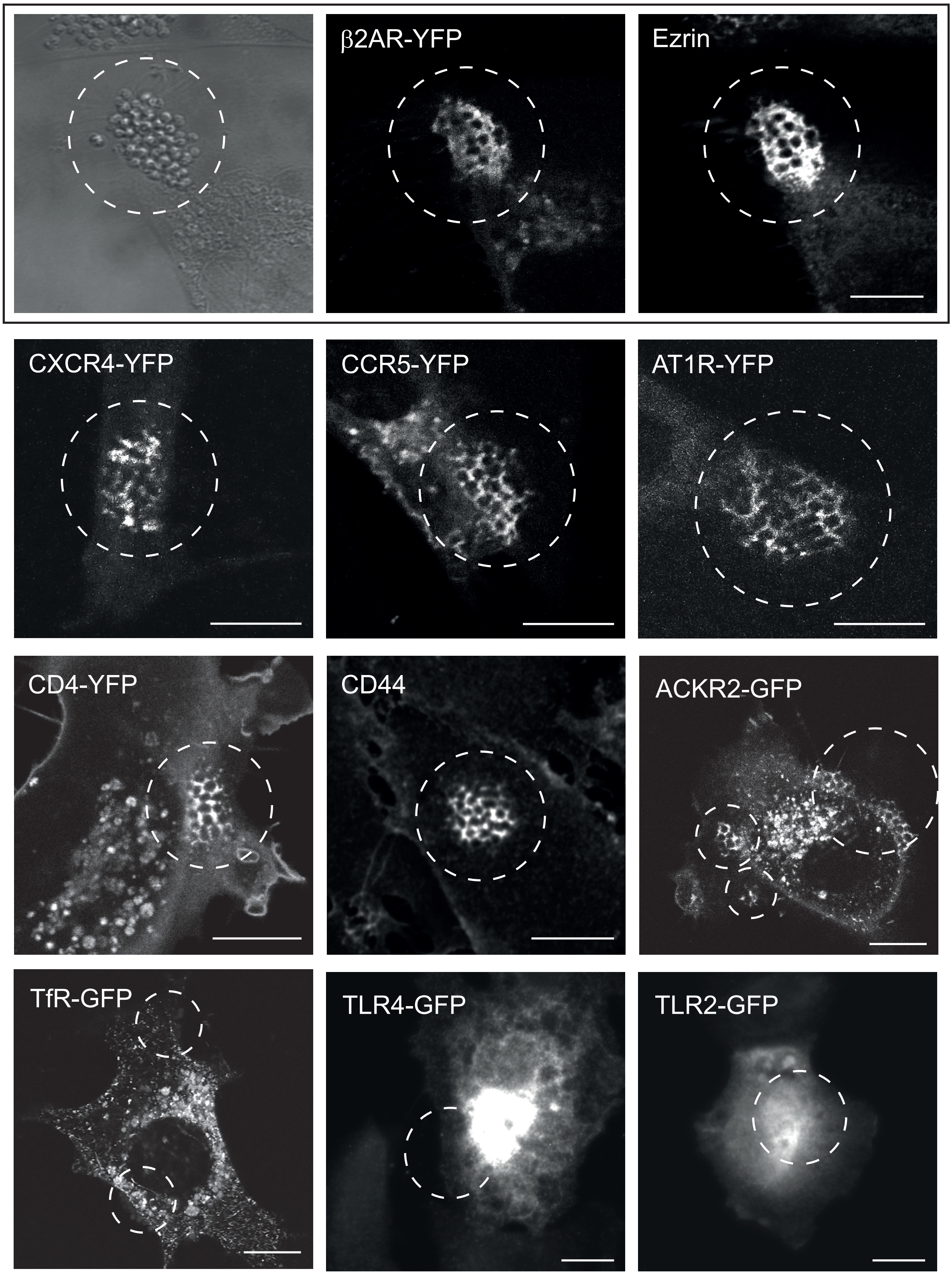

Supplementary figure 4: Selective recruitment of plasma membrane proteins to bacterial colonies

First raw: representative confocal microscopy and DIC images of a wild type *N. meningitidis* (strain 2C4.3) colony adhering to EA.hy926 endothelial cells (at least two independent experiments). The β2-adrenergic receptor tagged with YFP and Ezrin are both accumulated below a colony. Raw 2 to 4: representative microscopy images of EA.hy926 endothelial cells expressing CXCR4-YFP, CCD5, YFP, AT1R-YFP, CD4-YFP, Transferrin-receptor (TfR)-GFP, ACKR2-GFP (confocal microscopy), or TLR2-GFP, TLR4-GFP (apotome microscopy), or stained for endogenous CD44 (apotome microscopy). Scale bar 10μm (confocal microscopy) or 5 μm (apotome microscopy).

Supplementary figure 5

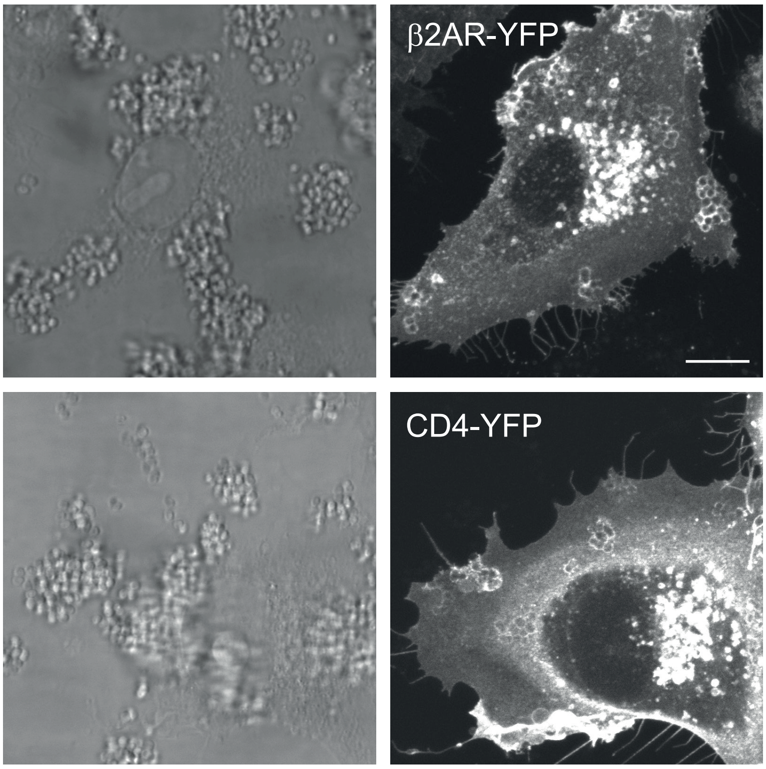

Supplementary figure 5: Receptor accumulation under colonies in fixed cells.
Representative DIC images and confocal microscopy images of fixed EA.hy926 endothelial cells expressing the β2-adrenergic receptor or CD4 tagged with YFP and infected with wild type *N. meningitidis* (strain 2C4.3). Two independent experiments. Z-stack projection. Scale bar 10μm

Supplementary figure 6

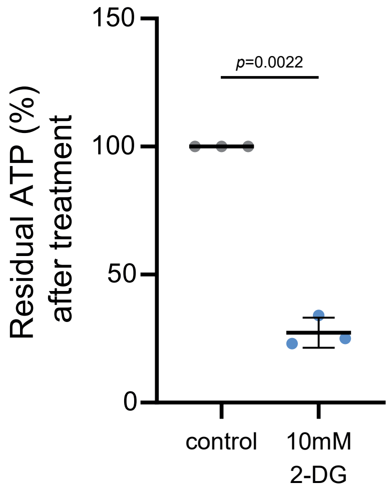

Supplementary figure 6: Measurement of ATP depletion
Complementary images from Figure 5A, B. Quantification of ATP concentration in cells treated (2-DG) with 10mM 2-DG or not (control). Three experiments were pooled. Data are presented as means % of control ± SD. One sample *t* test.
